# Atypical acute retinal necrosis accompanied by Terson’s syndrome: a case report

**DOI:** 10.1186/s12886-017-0655-4

**Published:** 2017-12-19

**Authors:** Jong Young Lee, Dong Yoon Kim, Hye Jin Lee, Jin Ho Jeong, Sung Pyo Park, Jin Young Kim

**Affiliations:** 1 Department of Ophthalmology, Jeju National University Hospital, Jeju National University School of Medicine, 1753-3 Ara-1 Dong, Jeju-Si, Jeju Self-Governing Province Republic of Korea; 2 Department of Ophthalmology, Chungbuk National University College of Medicine, Cheongju, South Korea; 3 Department of Ophthalmology, Kangdong Sacred Heart Hospital, Hallym University College of Medicine, Seoul, South Korea

**Keywords:** Acute retinal necrosis, Intravitreal ganciclovir injection, Terson’s syndrome

## Abstract

**Background:**

Acute retinal necrosis (ARN) has characterized by panuveitis, vitritis, severe vaso-occlusive vasculitis, and diffuse necrotizing retinitis. There are no case reports on atypical ARN combined with Terson’s syndrome. Herein, we report a case of ARN with atypical clinical features combined with Terson’s syndrome that we successfully treated by intravitreal ganciclovir injection.

**Case presentation:**

A 64-year-old man visited our eye clinic with a complaint of decreased visual acuity in his right eye. At the initial visit, his best corrected visual acuity was 20/125 in the right eye. Slit-lamp examination demonstrated mild hyperemia, keratic precipitates, and anterior chamber inflammatory reaction. Fundus examination revealed multiple diffuse white-yellowish infiltrations in the peripheral retina combined with dot hemorrhages. Ultra-wide-field fluorescence angiography showed obstructive arteritis with peripheral non-perfusion and leakage from the retinal vessels. As a result of the PCR analysis, varicella zoster virus DNA was identified in the aqueous humor. Under the diagnosis with VZV-mediated ARN, we started with intravenous acyclovir and oral prednisolone. After 3 days of the above treatment, the anterior chamber inflammation and vitreous opacity were increased. On fundus examination, multiple whitish infiltrations were increased. In addition, newly developed vitreous and peripapillary hemorrhages were detected. On the T2 brain magnetic resonance imaging (MRI) demonstrated a sub-acute or old hemorrhagic infarction in the right occipital lobe, and contrast-enhancing lesions in the right basal ganglia. The spinal tapping was performed in the department of neurology in our hospital at the time when the patient complained of headache, and intracranial pressure was 31 mmHg. Under the diagnosis of ARN with Terson’s syndrome, we started intravitreal ganciclovir (2 mg/0.5 ml) injections. After 5 intravitreal ganciclovir injections over a period of 8 months, the diffuse whitish infiltrating retinal lesions combined with dot hemorrhage were decreased. The vitreous and peripapillary hemorrhage was significantly reduced. There was no recurrence in the patient’s right eye, in which his visual acuity had improved to 20/60.

**Conclusions:**

In the event of a poor response to traditional treatment such as intravenous acyclovir, intravitreal ganciclovir may have a role as an adjunctive therapy in patients of VZV associated ARN combined with Terson’s syndrome.

## Background

Acute retinal necrosis (ARN) was first described in 1971 in a Japanese journal as being characterized by panuveitis, vitritis, severe vaso-occlusive vasculitis, and diffuse necrotizing retinitis. ARN preferentially affects the peripheral retina [1, 2]. The condition usually presents unilaterally, but bilateral cases have been described [3]. Most reported cases have been caused by varicella zoster virus (VZV), which accounts for 50%–80% of cases. However, herpes simplex virus (HSV) type 1 or 2 has also been the presumptive causative agent in some cases [4–6].

The diagnosis of ARN is usually based on clinical appearance and confirmation of causative viral DNA using serum or intraocular fluid antibody testing, viral culture, retinal biopsy, and polymerase chain reaction (PCR) [7, 8]. It is now more preferable to test focal samples rather than conducting a serum antibody titer for viruses. Use of polymerase chain reaction (PCR) system in aqueous humor and vitreous fluid samples is useful for identifying the viral origin. PCR is currently the preferred method of viral diagnosis [9–11].

The initial treatment for ARN is intravenous acyclovir (1500 mg/m^2^/day) for 5–10 days, followed by oral acyclovir (800 mg five times/day) for 4-6 weeks. Intravitreal foscanet or ganciclovir injection may also have a role as adjunctive therapy in the management of patients with ARN. Intravitreal foscarnet in combination with systemic antiviral therapy may reduce the risk of retinal detachment in VZV associated ARN [12, 13].

In this report, we describe an unusual case of ARN with atypical clinical features accompanied by Terson’s syndrome that was successfully treated by intravitreal ganciclovir injection.

## Case presentation

A 64-year-old man visited our eye clinic with a two-day history of decreased visual acuity in his right eye. The patient presented with a medical history of hypertension and diabetes mellitus. He stated that his vision had gradually worsened in recent days.

**Fig. 1:**
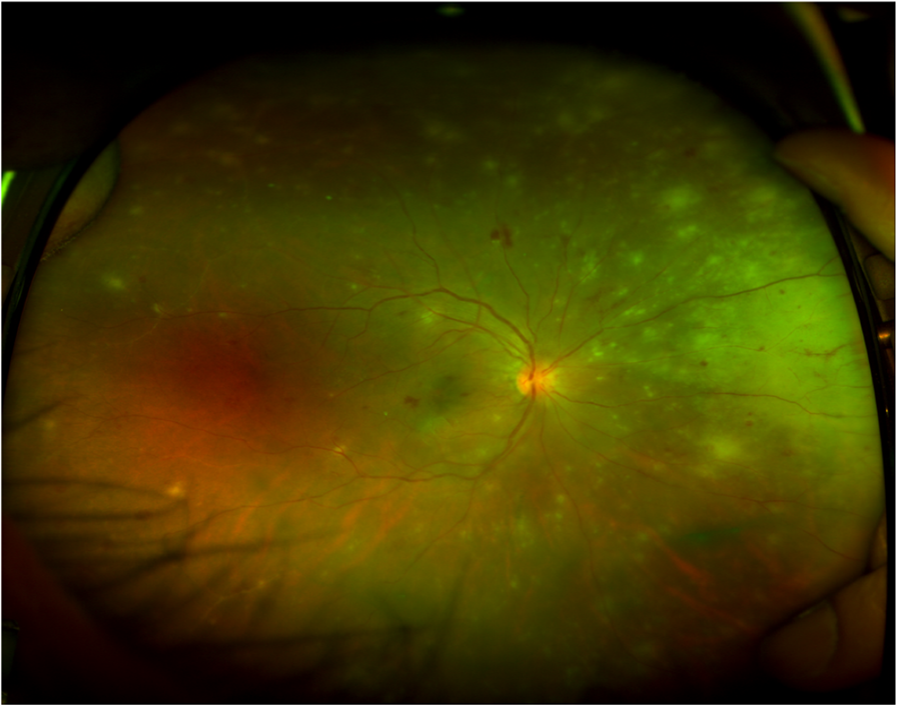
Ultra-wide-field photograph of the right eye at the initial visit. The mild vitritis with vasculitis and confluent retinitis in the nasal periphery are noted

**Fig. 2:**
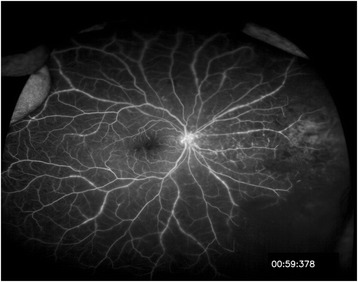
Ultra-wide-field fluorescence angiography showed diffuse arteriolar and venular occlusions at the initial visit. Hyperfluorescence is prominent in the nasal area

At the initial visit, his best-corrected visual acuity was 20/125 in the right eye and 20/25 in the left eye, and intraocular pressures were 18 mmHg and 13 mmHg, respectively. Slit-lamp examination demonstrated mild hyperemia and keratic precipitates. The eye also showed inflammation in the anterior chamber and anterior vitreous, which was scored as grade 2+ and grade 1+ according to the Standardization of Uveitis Nomenclature Working Group guidelines [14]. Fundus examination of the right eye showed multiple and diffuse white-yellowish infiltrations combined with retinal dot hemorrhages in the peripheral retina (Fig. 1). The left eye was completely normal. Ultra-wide-field fluorescence angiography showed obstructive retinal arteritis with peripheral non-perfusion and late leakage from the retinal vessels in the right eye (Fig. 2).

The patient was hospitalized for further evaluation and appropriate management. Basic laboratory test and serology study were performed. The serological tests were positive for HSV type 1 immunoglobulin G (IgG), VZV IgG, Epstein-Barr virus viral capsid antigen IgG, and Toxoplasma-specific IgG antibodies. Anterior chamber paracentesis and PCR analysis of the aqueous humor have been performed. VZV DNA was identified in the aqueous humor. But, DNA for HSV-1, HSV-2, and cytomegalovirus was not detected. Given the impression of VZV associated ARN, treatment was started with intravenous acyclovir (1200 mg three times a day). Oral prednisolone (1 mg/kg/day) was also started 24 h after systemic acyclovir treatment.

**Fig. 3:**
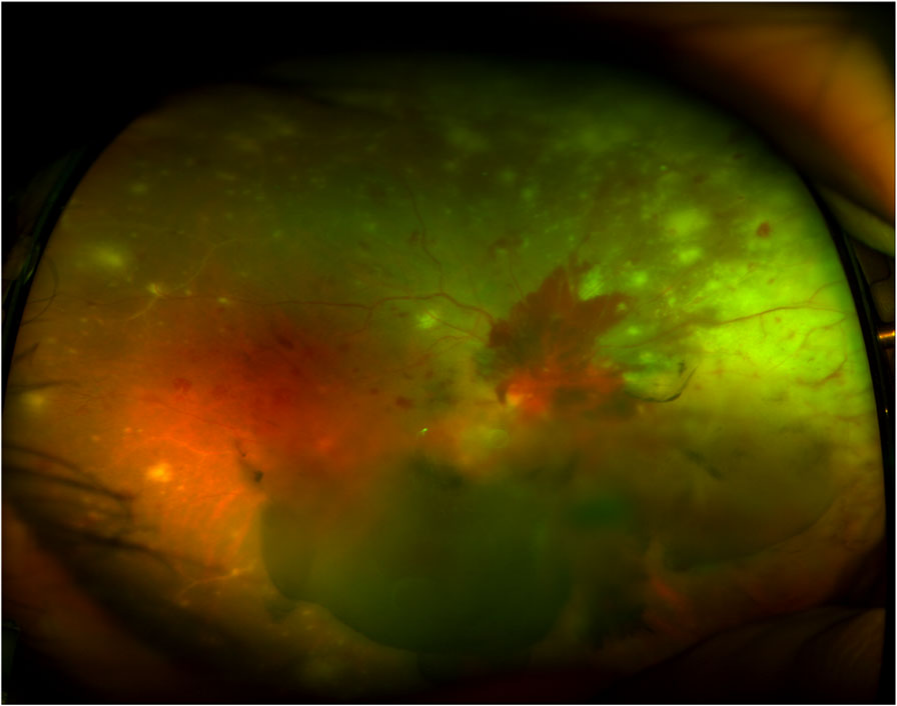
Ultra-wide-field photograph of the right eye at 3 days after admission. One of the whitish lesions was enlarged and had progressed to the posterior pole, accompanied by vitreous and peripapillary hemorrhage

**Fig. 4:**
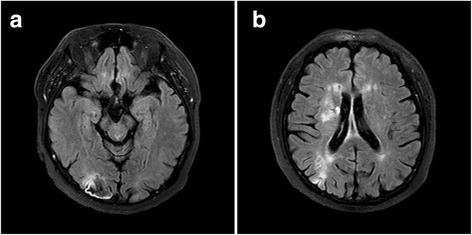
T2-weighted magnetic resonance images (**a**, **b**) of the brain. **a** A sub-acute or old hemorrhagic infarction in the right occipital lobe. **b** Contrast-enhancing lesions in the right basal ganglia that may be a cause of the suspected increase in intracranial pressure

After 3 days of the above treatment, slit-lamp examination revealed 3+ cells in the anterior chamber, 2+ cells in the anterior vitreous, and increased vitreous opacity (grade 2) in the right eye. On the fundus examination, the multiple whitish infiltrations were increased and newly developed vitreous and peripapillary hemorrhages were detected in the right eye (Fig. 3). At that time, the patient had symptoms of headache. T2-weighted MRIs of the brain performed to find the cause of the atypical ARN demonstrated a sub-acute or old hemorrhagic infarction in the right occipital lobe, and contrast-enhancing lesions in the right basal ganglia (Fig. 4a and b). The spinal tapping was performed in the department of neurology in our hospital at the time when the patient complained of headache, and intracranial pressure was 31 mmHg. This suggests that the possibility of Terson’s syndrome due to suddenly increased intracranial pressure in this case [15].

**Fig. 5:**
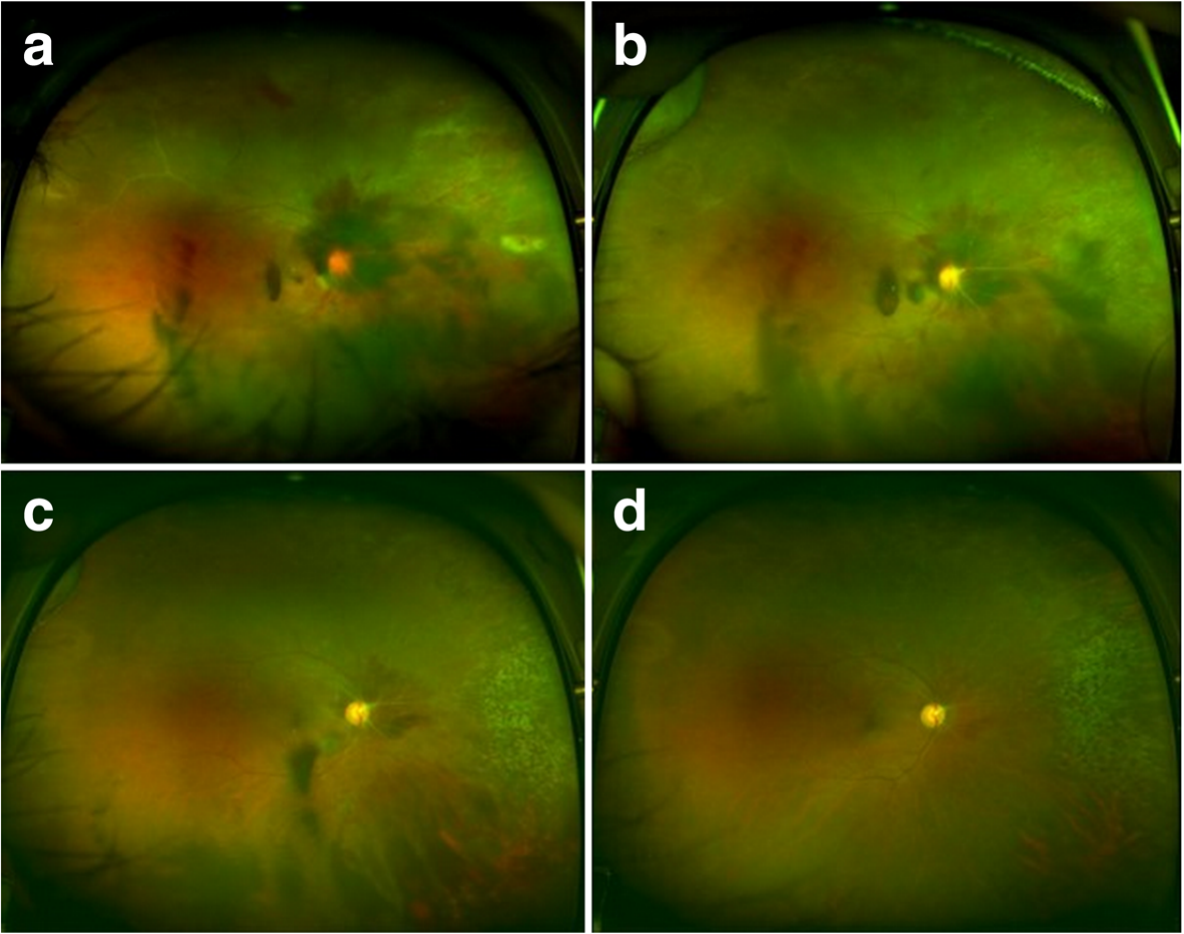
Regression of atypical retinal necrosis in the right eye (**a**, **b**, **c**, **d**). **a** On day 11, the whitish retinal lesion was decreased. **b**, **c** At 1 and 3 months after the initial visit, the peripheral retinal necrosis and vitreous hemorrhage have decreased. **d** Eight months after the first visit, the vitreous and peripapillary hemorrhage are nearly absorbed. Peripheral retinal necrosis is not detected

In view of the patient’s clinical features, immune status, and brain images, he was diagnosed as having VZV-mediated ARN with Terson’s syndrome. On day 5 of the admission, the patient’s visual acuity was reduced to counting fingers at 50 cm in the right eye. Retinal infiltration and vitreous hemorrhage were increased. Therefore, we started intravitreal injections of ganciclovir (2 mg/0.5 mL). After 5 intravitreal injections (from the 5 to 15 day of the admission), the multiple white-yellowish infiltrative lesions and retinal dot hemorrhages were decreased, and visual acuity increased to 20/800 in the right eye. The vitreous and peripapillary hemorrhages were also markedly decreased compared with the initial status of the ARN. After 20 days of intravenous antiviral therapy, the patient was discharged on oral valacyclovir, 1000 mg twice a day, combined with an oral prednisolone (tapering dose, 0.5 m/kg/day). After 5 intravitreal ganciclovir injections over a period of 8 months, there was no recurrence in the patient’s right eye, in which his visual acuity had improved to 20/60 (Fig. 5a and b). The contralateral eye remained normal.

## Discussion and conclusions

ARN is a rare vision-threatening disease characterized by rapidly progressive, peripheral retinal necrosis combined with inflammation of the vitreous or anterior chamber. Traditional treatment of ARN is induction with intravenous acyclovir followed by oral acyclovir. [12]. Here we have described a case of ARN with atypical clinical features that progressed to retinal necrosis accompanied by vitreous and peripapillary hemorrhage. Despite of aggressive treatment with systemic antiviral agent and steroids, the retinal necrosis progressed and a sub-acute or old hemorrhagic infarction was detected on MRI of the brain. The spinal tapping was performed in the department of neurology in our hospital at the time when the patient complained of headache, and intracranial pressure was 31 mmHg. The patient was diagnosed with VZV-mediated ARN combined with Terson’s syndrome. The multiple whitish, infiltrating retinal lesions combined with peripapillary hemorrhage responded well to intravitreal ganciclovir injections. To our knowledge, there are no case reports on atypical ARN accompanied by Terson’s syndrome. We also found that in the event of a poor response to traditional treatment such as intravenous acyclovir, intravitreal ganciclovir may have a role as an adjunctive therapy in patients of VZV associated ARN combined with Terson’s syndrome.

## Abbreviations

ANR: Acute retinal necrosis
HSV: Herpes simplex virus
IgG: Immunoglobulin G
MRI: Brain magnetic resonance imaging
PCR: Polymerase chain reaction
VZV: Varicella zoster virus
